# COVLIAS 1.0: Lung Segmentation in COVID-19 Computed Tomography Scans Using Hybrid Deep Learning Artificial Intelligence Models

**DOI:** 10.3390/diagnostics11081405

**Published:** 2021-08-04

**Authors:** Jasjit S. Suri, Sushant Agarwal, Rajesh Pathak, Vedmanvitha Ketireddy, Marta Columbu, Luca Saba, Suneet K. Gupta, Gavino Faa, Inder M. Singh, Monika Turk, Paramjit S. Chadha, Amer M. Johri, Narendra N. Khanna, Klaudija Viskovic, Sophie Mavrogeni, John R. Laird, Gyan Pareek, Martin Miner, David W. Sobel, Antonella Balestrieri, Petros P. Sfikakis, George Tsoulfas, Athanasios Protogerou, Durga Prasanna Misra, Vikas Agarwal, George D. Kitas, Jagjit S. Teji, Mustafa Al-Maini, Surinder K. Dhanjil, Andrew Nicolaides, Aditya Sharma, Vijay Rathore, Mostafa Fatemi, Azra Alizad, Pudukode R. Krishnan, Nagy Frence, Zoltan Ruzsa, Archna Gupta, Subbaram Naidu, Mannudeep Kalra

**Affiliations:** 1 Stroke Diagnostic and Monitoring Division, AtheroPoint™, Roseville, CA 95661, USA; drindersingh1@gmail.com (I.M.S.); pomchadha@gmail.com (P.S.C.); 2 Advanced Knowledge Engineering Centre, GBTI, Roseville, CA 95661, USA; sushant.ag09@gmail.com; 3 Department of Computer Science Engineering, PSIT, Kanpur 209305, India; 4 Department of Computer Science Engineering, Rawatpura Sarkar University, Raipur 492015, India; drrkpathak20@gmail.com; 5 Mira Loma High School, Sacramento, CA 95821, USA; manvi.ketireddy@gmail.com; 6 Department of Radiology, Azienda Ospedaliero Universitaria (A.O.U.), 09124 Cagliari, Italy; martagiuliacol@gmail.com (M.C.); lucasabamd@gmail.com (L.S.); antonellabalestrieri@hotmail.com (A.B.); 7 Department of Computer Science, Bennett University, Noida 201310, India; suneet.gupta@bennett.edu.in; 8 Department of Pathology—AOU of Cagliari, 09124 Cagliari, Italy; gavinofaa@gmail.com; 9 The Hanse-Wissenschaftskolleg Institute for Advanced Study, 27753 Delmenhorst, Germany; monika.turk84@gmail.com; 10 Department of Medicine, Division of Cardiology, Queen’s University, Kingston, ON K7L 3N6, Canada; johria@queensu.ca; 11 Department of Cardiology, Indraprastha APOLLO Hospitals, New Delhi 208011, India; drnnkhanna@gmail.com; 12 Department of Radiology, University Hospital for Infectious Diseases, 10000 Zagreb, Croatia; klaudija.viskovic@bfm.hr; 13 Cardiology Clinic, Onassis Cardiac Surgery Center, 176 74 Athens, Greece; soma13@otenet.gr; 14 Heart and Vascular Institute, Adventist Health St. Helena, St. Helena, CA 94574, USA; Lairdjr@ah.org; 15 Minimally Invasive Urology Institute, Brown University, Providence City, RI 02912, USA; gyan_pareek@brown.edu (G.P.); dwsobel@gmail.com (D.W.S.); 16 Men’s Health Center, Miriam Hospital Providence, Providence, RI 02906, USA; martin_miner@brown.edu; 17 Rheumatology Unit, National Kapodistrian University of Athens, 157 72 Athens, Greece; psfikakis@med.uoa.gr; 18 Department of Transplantation Surgery, Aristoteleion University of Thessaloniki, 541 24 Thessaloniki, Greece; tsoulfasg@gmail.com; 19 National & Kapodistrian University of Athens, 157 72 Athens, Greece; aprotog@med.uoa.gr; 20 Department of Immunology, Sanjay Gandhi Postgraduate Institute of Medical Sciences, Lucknow 226014, India; durgapmisra@gmail.com (D.P.M.); vikasagr@yahoo.com (V.A.); 21 Academic Affairs, Dudley Group NHS Foundation Trust, Dudley DY1 2HQ, UK; george.kitas@nhs.net; 22 Arthritis Research UK Epidemiology Unit, Manchester University, Manchester M13 9PL, UK; 23 Ann and Robert H. Lurie Children’s Hospital of Chicago, Chicago, IL 60611, USA; jteji@mercy-chicago.org; 24 Allergy, Clinical Immunology and Rheumatology Institute, Toronto, ON M5G 1N8, Canada; almaini@hotmail.com; 25 Athero Point LLC, Roseville, CA 95611, USA; surinderdhanjil@gmail.com (S.K.D.); Vijay.s.rathore@kp.org (V.R.); 26 Vascular Screening and Diagnostic Centre, University of Nicosia Medical School, Nicosia 2408, Cyprus; anicolaides1@gmail.com; 27 Division of Cardiovascular Medicine, University of Virginia, Charlottesville, VA 22904, USA; AS8AH@hscmail.mcc.virginia.edu; 28 Department of Physiology & Biomedical Engg., Mayo Clinic College of Medicine and Science, Rochester, MN 55905, USA; fatemi.mostafa@mayo.edu; 29 Department of Radiology, Mayo Clinic College of Medicine and Science, Rochester, MN 55905, USA; aza@mayo.edu; 30 Neurology Department, Fortis Hospital, Bangalore 560076, India; prkrish12@rediffmail.com; 31 Department of Internal Medicines, Invasive Cardiology Division, University of Szeged, 6720 Szeged, Hungary; drnagytfer@hotmail.com (N.F.); zruzsa@icloud.com (Z.R.); 32 Radiology Department, Sanjay Gandhi Postgraduate Institute of Medical Sciences, Lucknow 226014, India; garchna@gmail.com; 33 Electrical Engineering Department, University of Minnesota, Duluth, MN 55455, USA; dsnaidu@d.umn.edu; 34 Department of Radiology, Massachusetts General Hospital, 55 Fruit Street, Boston, MA 02114, USA; mkalra@mgh.harvard.edu

**Keywords:** COVID-19, computed tomography, lungs, segmentation, hybrid deep learning

## Abstract

Background: COVID-19 lung segmentation using Computed Tomography (CT) scans is important for the diagnosis of lung severity. The process of automated lung segmentation is challenging due to (a) CT radiation dosage and (b) ground-glass opacities caused by COVID-19. The lung segmentation methodologies proposed in 2020 were semi- or automated but not reliable, accurate, and user-friendly. The proposed study presents a COVID Lung Image Analysis System (COVLIAS 1.0, AtheroPoint™, Roseville, CA, USA) consisting of hybrid deep learning (HDL) models for lung segmentation. Methodology: The COVLIAS 1.0 consists of three methods based on solo deep learning (SDL) or hybrid deep learning (HDL). SegNet is proposed in the SDL category while VGG-SegNet and ResNet-SegNet are designed under the HDL paradigm. The three proposed AI approaches were benchmarked against the National Institute of Health (NIH)-based conventional segmentation model using fuzzy-connectedness. A cross-validation protocol with a 40:60 ratio between training and testing was designed, with 10% validation data. The ground truth (GT) was manually traced by a radiologist trained personnel. For performance evaluation, nine different criteria were selected to perform the evaluation of SDL or HDL lung segmentation regions and lungs long axis against GT. Results: Using the database of 5000 chest CT images (from 72 patients), COVLIAS 1.0 yielded AUC of **~0.96, ~0.97, ~0.98**, and **~0.96** (*p*-value < 0.001), respectively within 5% range of GT area, for SegNet, VGG-SegNet, ResNet-SegNet, and NIH. The mean Figure of Merit using four models (left and right lung) was above **94%**. On benchmarking against the National Institute of Health (NIH) segmentation method, the proposed model demonstrated a **58%** and **44%** improvement in ResNet-SegNet, **52%** and **36%** improvement in VGG-SegNet for lung area, and lung long axis, respectively. The PE statistics performance was in the following order: **ResNet-SegNet > VGG-SegNet > NIH > SegNet**. The HDL runs in <1 s on test data per image. Conclusions: The COVLIAS 1.0 system can be applied in real-time for radiology-based clinical settings.

## 1. Introduction

On 30 January 2020, the International Health Regulations and Emergency Committee of the World Health Organization (WHO) declared COVID-19 or Coronavirus a “public health emergency of international concern” or “pandemic”. As per the WHO’s statistics dated 28 July 2021, more than **196** million people have been infected with Acute Respiratory Distress Syndrome (ARDS), and nearly **4.2** million have lost their lives due to this virus [1]. This SARS-CoV-2 virus affects the respiratory system, damages lungs, travels through the entire body, and causes myocardial infarction or coronary artery syndrome [2,3] or worsening diabetes [4] or causing pulmonary embolism [5]. It was seen that comorbidity had a severe influence on COVID-19 [6]. As of today, even though vaccination is showing the signs of pandemic control in some parts of the world, it is still the highest concern for health professionals. The objective is to flatten the curve and evaluate the COVID-19 lung severity at an early stage prior to worsening symptoms and admission of patients in ICU [7].

Computed Tomography (CT) is often used for assessing COVID-19 severity in the lung regions and is considered an important component of the computer-aided diagnosis for lung image analysis [7,8,9]. For a complete diagnosis of the COVID-19 severity, one must first identify the region of interest in these CT scans. There are two main challenges associated with the processing of CT scans: first and foremost, the challenge is the large volume of patients in diagnostic centers with each having 200 slices to be processed. This makes the task of processing scanned images tedious and time-consuming [10]. The second issue with current automated or semi-automated systems is reliability, accuracy, and clinical effectiveness [11]. One of the major causes for unreliable accuracy and low performance is the intensity-based segmentation methods which are influenced by local or global statistical methods. Furthermore, it does not take advantage of the cohort’s knowledge. Thus, there is a clear need for an automated and accurate joint left and right lung identification system in CT scans.

Artificial Intelligence (AI) has provided fast pace research and development in nearly all walks of life especially in healthcare imaging [12]. The reason for such growth is the ability of AI techniques to mimic the brain using deep neural networks [12,13,14]. Machine learning is the most fundamental class of AI and has dominated the medical imaging industry [15], covering many fields of healthcare such as cancer classification and diagnosis of several kinds of cancers such as breast [16], liver [17,18], thyroid [19,20,21], skin [22,23], ovarian [24], and lung [25]. ML has also been applied to risk stratification for coronary artery disease [26,27], atherosclerosis imaging [28,29], and stroke [30], diabetes [31,32], and arrhythmia classification [33].

A subclass of ML is deep learning (DL), or conventionally called solo DL (SDL). Many researchers have proposed AI-based studies on COVD-19 which involves SDL techniques. [34,35]. It offers substantial benefits compared to ML-based solutions [11,36]. DL provides a complete automated feature extraction and classification simultaneously using the so-called dense layers. Despite the merits of DL, it poses challenges and uncertainties such as optimization of the learning rate, deciding the number of epochs, avoiding overfitting, handling large image size configurations, and operating in a multiresolution framework. One way to overcome challenges in SDL is by fusing two kinds of DL systems, called hybrid DL (HDL). It offers the advantages such as (i) extraction and refinement of a large number of features, (ii) application of diversity in classifiers, and (iii) ability to apply transfer learning for weights reusability. The proposed study uses the spirit of HDL to design COVID Lung Image Analysis System (COVLIAS) 1.0, which computes the segmentation of the left and right lungs jointly. The proposed framework uses two kinds of HDL models which combine Visual Geometry Group (VGG), Segmentation Network (SegNet), and Residual Network (ResNet) architectures. The model combines the strengths of both VGG and SegNet models and offers a new VGG-SegNet framework.

Similarly, Resnet-Segnet includes the power of both ResNet and SegNet. These models use 40:60 training to testing ratio protocol, then benchmarking our novel design against the NIH-based Fuzzy-Connectedness (FC) lung segmentation method [37]. Finally, we compute nine different ways for conducting the performance evaluation of the lung region and boundary estimation system.

**Figure 1:**
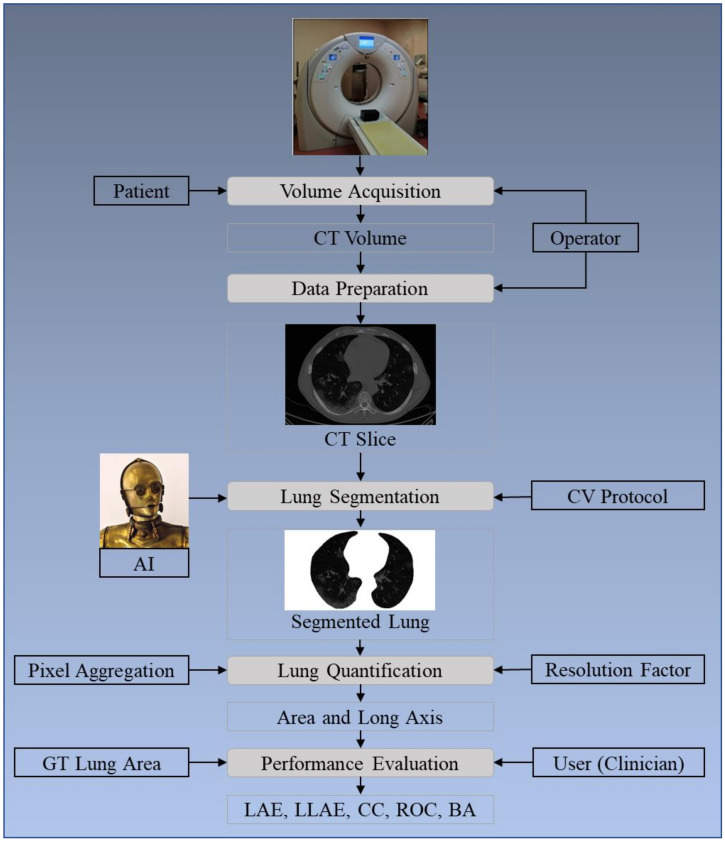
COVLIAS 1.0: Global system for lung segmentation and evaluation. AI: Artificial Intelligence; CV: Cross-Validation; GT: Ground Truth; LAE: Lung Area Error; LLAE: Lung Long Axis Error; CC: Correlation coefficient; ROC: Receiver operating characteristic; BA: Bland–Altman.

The layout of this study is as follows: Section 2 presents the methodology with the architecture of the COVLIAS 1.0 (Figure 1) using HDL and SDL. The experimental protocol is shown in Section 3, while results and comprehensive performance evaluation are presented in Section 4. The discussions and benchmarking are presented in Section 5. Finally, the paper concludes in Section 6.

## 2. Methodology

### 2.1. Patient Demographics, Image Acquisition, and Data Preparation

#### 2.1.1. Demographics

The dataset consists of 72 Italian patients, where 46 patients were male and 26 were female. The mean height and weight were 172.72 cm and 79 kg, respectively. A total of 60 patients were tested for PCR, while 12 could not be tested for PCR.

#### 2.1.2. Image Acquisition

**Figure 2:**
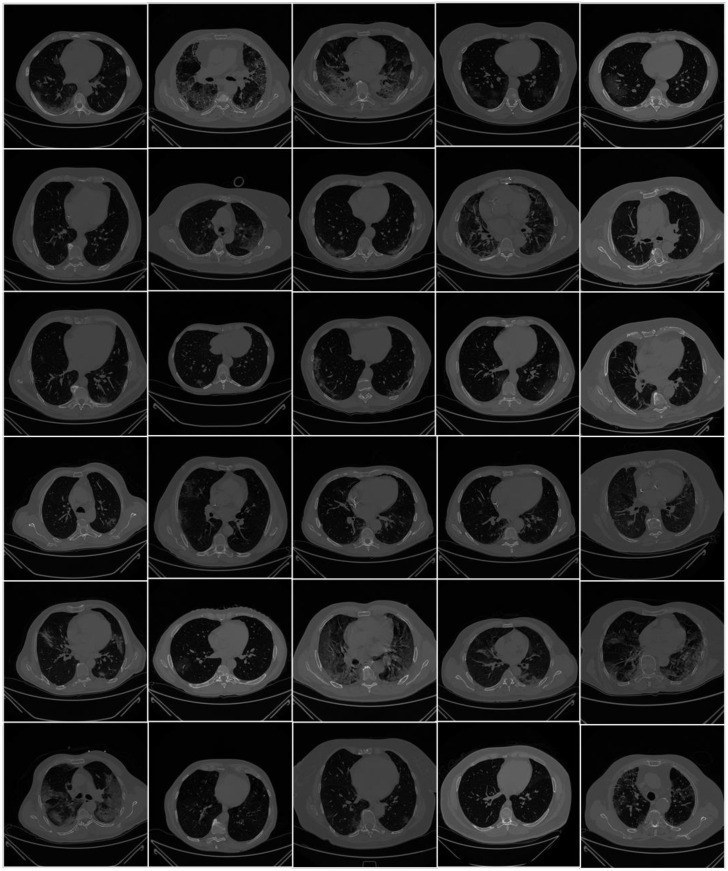
Raw lung COVID-19 CT images were taken from different patients in the database.

All chest computed tomography (CT) scans were performed during a single full inspiratory breath-hold in a supine position on a 128-slice multidetector-row CT scanner (Philips Ingenuity Core, Philips Healthcare, the Netherlands). No intravenous or oral contrast media were administered. The CT examinations were performed at 120 kV, 226 mAs (using automatic tube current modulation—Z-DOM, Philips), with 1.08 spiral pitch factor, 0.5-s gantry rotation time, and 64 * 0.625 detector configurations. One-mm thick images were reconstructed with soft tissue kernel using 512 × 512 matrix (mediastinal window) and lung kernel using 768 × 768 matrix (lung window). CT images were reviewed on the Picture Archiving and Communication System (PACS) workstation equipped with two 35 × 43 cm monitors produced by Eizo, with a 2048 × 1536 matrix. Figure 2 shows the raw sample CT scans of COVID-19 patients with varying lung sizes and variable intensity patterns, posing a challenge.

#### 2.1.3. Data Preparation

The CT data of 72 COVID-positive patients’ data are used in the proposed study. Each patient consisted of 200 slices, out of which 65–70 slices consisting of visible lung region were selected by the radiologist [LS]. A total of 5000 images were used in the proposed study. A total of 2000 images were used to train the AI-based segmentation models, whereas 3000 images were used to test and validate the models.

**Figure 3:**
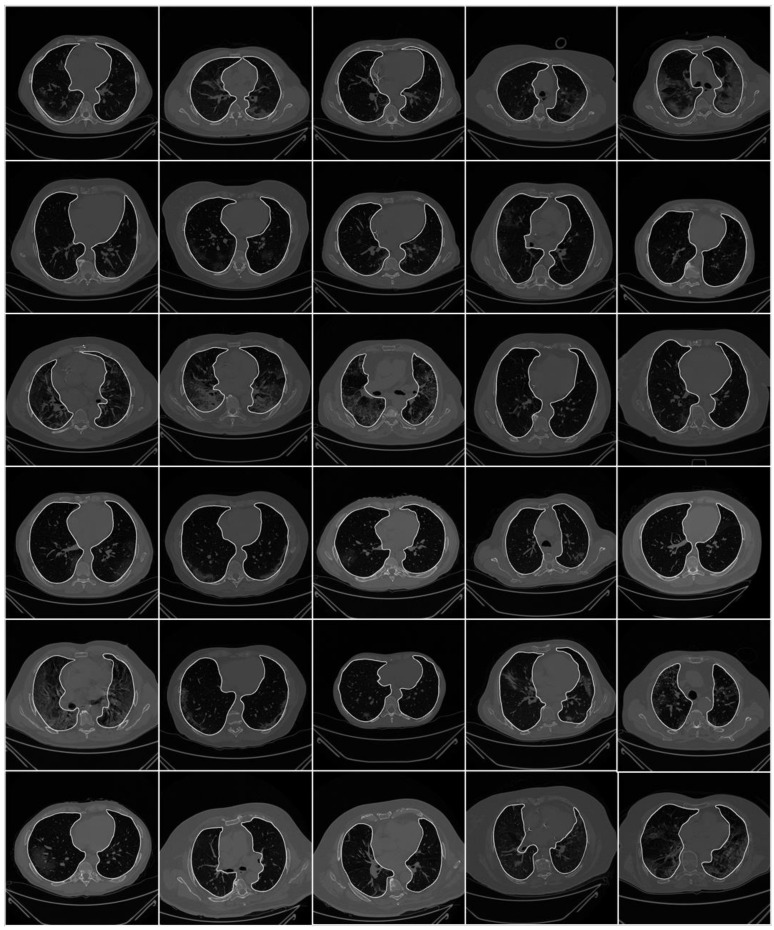
Raw lung images with ground truth boundary (white) overlay on grayscale CT scans.

**Figure 4:**
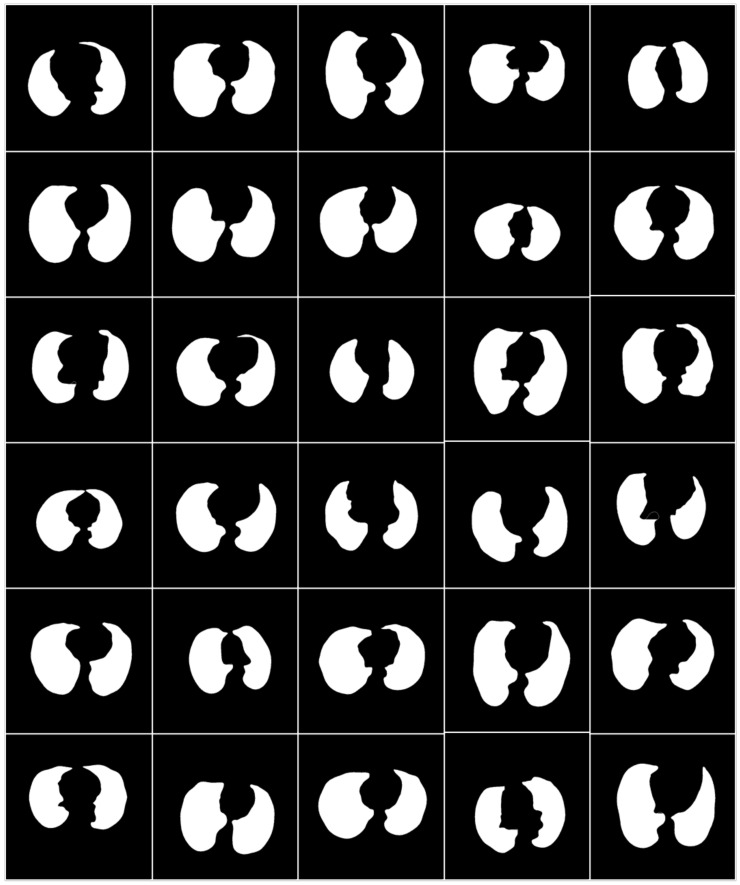
White binary mask of lung region used for AI-based model training.

To prepare the data for segmentation, a binary mask was generated manually by tracing in the selected slices under the guidance of a trained radiologist [LS] using ImgTracer™ (Global Biomedical Technologies, Inc., Roseville, CA, USA) [38,39,40]. Figure 3 shows the raw lung COVID-19 CT scans with ground truth boundary (white) overlay using manual tracing. Figure 4 shows the white binary mask of the lung region computed using manual ImgTracer™.

### 2.2. Architecture

The COVLIAS 1.0 system incorporates three models: deep learning-based (SDL) and two hybrid-based (HDL). We benchmark these models against the conventional NIH-based model using fuzzy connectedness. In all, the proposed study incorporates four kinds of models such as (a) SegNet, (b) VGG-SegNet, (c) ResNet-SegNet, and (d) NIH-conventional model.

#### 2.2.1. SegNet

**Figure 5:**
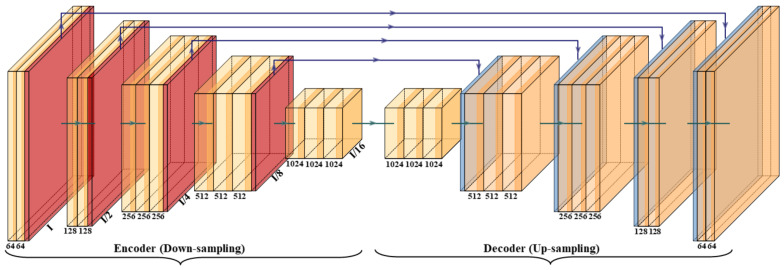
SegNet architecture.

SegNet is a semantic segmentation model shown in Figure 5 and has been implemented by Badrinarayanan et al. before [41], while we adapted SegNet for comparison with HDL. SegNet is a trainable segmentation architecture that consists of (i) an encoder network (the left half of Figure 5), (ii) a corresponding decoder network followed by (the right half of Figure 5) (iii) a pixel-wise classification layer (the last block of Figure 5). The architecture of the encoder network is topologically identical to the 13 convolutional layers. It uses a technique to down-sample encoder output, which involves storing the max-pooling (filter size of 2 × 2) indices. The max-pooling layer is placed at the end of each block on the encoder side, which increases the depth of the following layer by two times (64–128–256–512).

Similarly, in the second half of the architecture, the up-sampling happens where the depth of the layer decreases by two (512–256–128–64). The up-sampling process involves recalling the max-pooling indices at the corresponding encoder layer. Finally, in the end, there is a K-class softmax classifier used to predict the class for each pixel. This gives reasonably good performance and is space-efficient.

#### 2.2.2. VGG-SegNet

**Figure 6:**
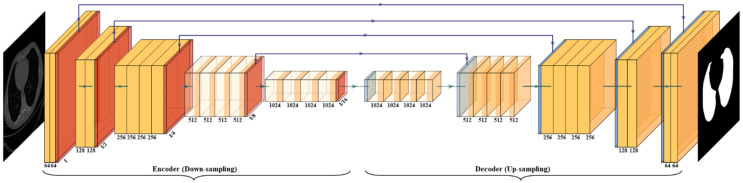
VGG-SegNet architecture.

The VGG architecture consists of blocks, where each block is composed of 2D convolution (Conv) and max-pooling layers (Figure 6). VGGNet was born out of the need to reduce the # of parameters in the convolution layers and improve training time [42]. This architecture consists of 16 convolution layers compared to 13 in SegNet. It helps the model to learn high-level features to improve the results. The blocks used in the VGG-SegNet architecture consist of (i) encoder, (ii) decoder part, and followed by (iii) a pixel-wise softmax classifier.

The distribution of the blocks in the encoder (the left half of Figure 6) is 2 × (2 Conv + max-pool) + 3 × (4 Conv + max-pool), and the decoder (the right half of Figure 6) is the reverse of the encoder part with up-sampling, thereby decreasing the depth of the layers.

#### 2.2.3. ResNet-SegNet

**Figure 7:**
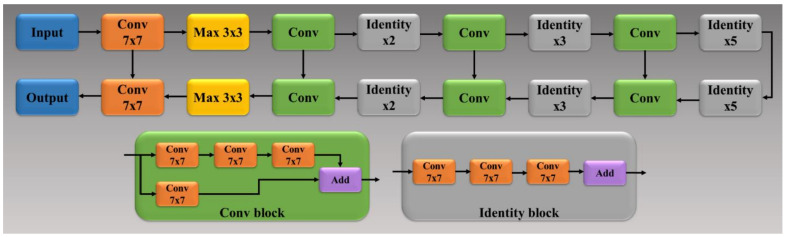
ResNet-SegNet architecture.

Residual Network (ResNet) [43] came into existence by overcoming the VGG architecture problem of the vanishing gradient. When the network goes through backpropagation, the gradient keeps flowing backward to the initial layers. This value keeps getting multiplied by each gradient. As a result, the gradient becomes smaller and smaller, making the updates to the initial layers very small, also known as the vanishing gradient problem. Consequently, this results in an increase of training time. ResNet is a superior architecture to VGG because it has skip connections, which act as gradient superhighways or bypasses, allowing the gradient to flow unhindered. This solves the problem of diminishing gradient. With the help of the identity function (shown in the bottom right of Figure 7), the value of backpropagated gradient values does not decrease because the local gradient value is one.

#### 2.2.4. NIH Segmentation Model

**Figure 8:**
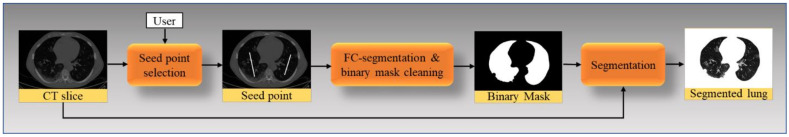
User interactive fuzzy-connectedness Center for Infectious Disease Imaging (CIDI) lung segmentation [37].

Figure 8 shows the architecture of the fuzzy-connected system for segmentation of the lung region. This system was not built in the study but simply adapted from the NIH website [37] for benchmarking. There are three steps in the process: (i) seed point selection, (ii) fuzzy-connected (FC) segmentation and binary mask cleaning, and (iii) grayscale segmentation. The user can select the seed point in two lines corresponding to the left and right lungs. FC segmentation is executed, and the results are displayed in the form of a binary mask. Finally, the lung is segmented with the given binary mask and the original CT COVID-19 grayscale scan leading to segmented lungs.

#### 2.2.5. Loss Functions for SDL and HDL Models

The proposed system uses CE-loss in SDL/HDL models. The CE-loss function, labeled as $L_{CE}$ for all the models, can be mathematically given by Equation (1):

(1) $$L_{CE}{=-[(y}_{i}{\times \log\;a}_{i}{)+(1-y}_{i}{)\times \log(1-a}_{i})]$$

where y_i_ is the input GT label 1, (1 − y_i_) is GT label 0, a_i_ represents the softmax classifier probability, and x represents the product of the two terms. All three of the AI architectures shown in Figure 5, Figure 6 and Figure 7 have been trained using the CE-loss function.

### 2.3. Sample Size Calculation

It is vital to understand the sample size needed for achieving the desired performance. Generally, sample size requires calculating the number of studies adapted in our proposed system for reaching the desired accuracy and error statistics. This is crucial because, when the sample size is too large with respect to the desired sample size, it may incur more computational and economic burdens both in terms of time and cost. We follow paradigms like our previous paradigms for sample size calculation [27,44,45,46,47,48,49]. To represent the mathematical formula for the sample size calculation, we used a z-score from the standard z-table as shown by Equation (2):

(2) $$N_{p}={\left(z\right)}^{2}\times \frac{\hat{p}\left(1-\hat{p}\right)}{{\left(\mathrm{MoE}\right)}^{2}}\;$$

In the proposed study, we use a confidence level of 95%, a margin of error (MoE) of 5%, and a data proportion ($\hat{p}$) of 0.5. Normal distribution was assumed for the data sample, and a confidence interval of 95% provided a *z*-score value of 1.96 (derived from the standard z-table). MoE of 5% for SDL and 3% for HDL was used to ensure that the population remains in the tolerance band. The desired sample size (n) for a large, true population size was computed using Equation (2). Using the confidence level of 95%, the resultant sample size resulted in 1351 samples while using MoE of 3% for HDL and 588 samples for SDL with 5% MoE. Thus, the recruited sample size for HDL and SDL was ~270% and ~750% higher than the desired sample size.

## 3. Experimental Protocol

### 3.1. Cross-Validation 

A total of 5000 COVID-19 CT slices are used in the proposed system. A well-known data partitioning method, K5-r46, was used in our system, in which the database was partitioned into five parts (K5). Then, a 4:6 ratio was used for training to test, these 40% of (2000 images) data are used for training, and 60% (3000 images) of data are used for testing. An internal validation system was also used in our system with training data. For each test patient, the binary mask was generated, and the lung area was estimated.

### 3.2. Lung Quantification

The lung area is measured by counting the number of white pixels in the lung region and converted to mm^2^ dimensions using the resolution factor of 0.05 mm to a pixel. If *N* represents the total number of the image in the database, $A_{ai}\left(m,n\right)$ represents lung area for in the image “*n*” using the AI model “*m*”, ${\bar{A}}_{ai}\left(m\right)$ represents the mean lung area corresponding to the AI model “*m*”,$\;{\bar{A}}_{gt}$ represents the corresponding mean area of the GT binary lung mask, then mathematically ${\bar{A}}_{ai}\left(m\right)$ and ${\bar{A}}_{gt}$ can be computed and are shown in Equation (3):

(3) $$\left.\begin{array}{c} {\bar{A}}_{ai}\left(m\right)=\frac{\sum_{n=1}^{N}A_{ai}\left(m,n\right)}{N}\;\\ \;{\bar{A}}_{gt}=\frac{\sum_{n=1}^{N}A_{gt}\left(n\right)}{N} \end{array}\right\}$$

### 3.3. Accuracy Computation

The accuracy of the AI system is measured by comparing the predicted output and the ground truth pixel values. These values were interpreted as binary (0 or 1) numbers as the output lung mask was only black and white, which was then summed up and divided by the total number of pixels. Assuming TP represents true positive, TN represents true negative, FN represents false negative, and FP represents false positive. The accuracy of the AI system can be given as shown in Equation (4) [50]:

(4) $$\mathrm{ACC}\;\left(\%\right)=\left(\frac{\mathrm{TP}+\mathrm{TN}}{\mathrm{TP}+\mathrm{FN}+\mathrm{TN}+\mathrm{FP}}\right)\times 100$$

## 4. Results and Performance Evaluation

### 4.1. Results

**Figure 9:**
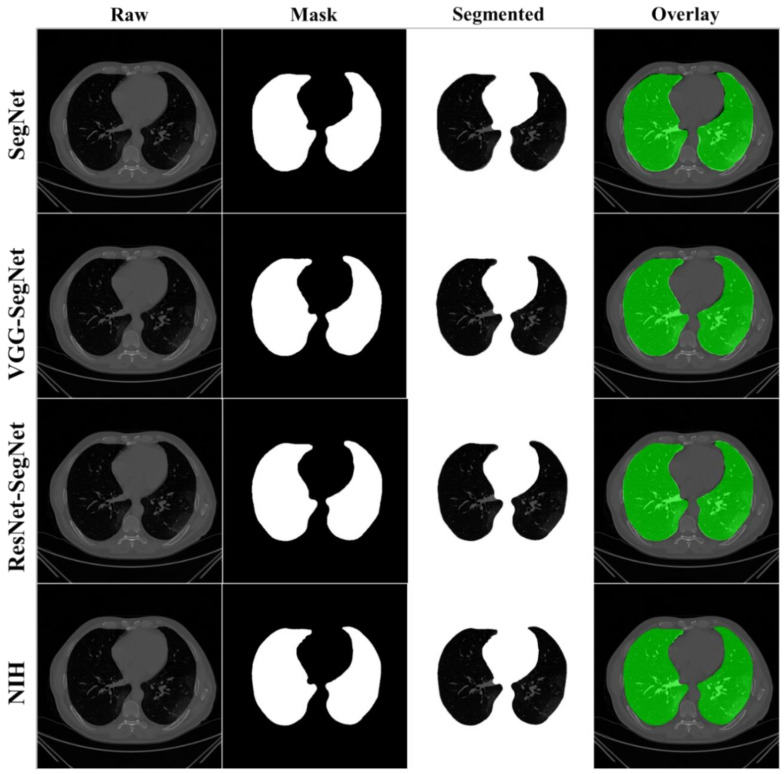
Results from the four AI models with grayscale CT slice. Row 1 to Row 4 is four models: SegNet, VGG-SegNet, ResNet-SegNet, and NIH.

**Figure 10:**
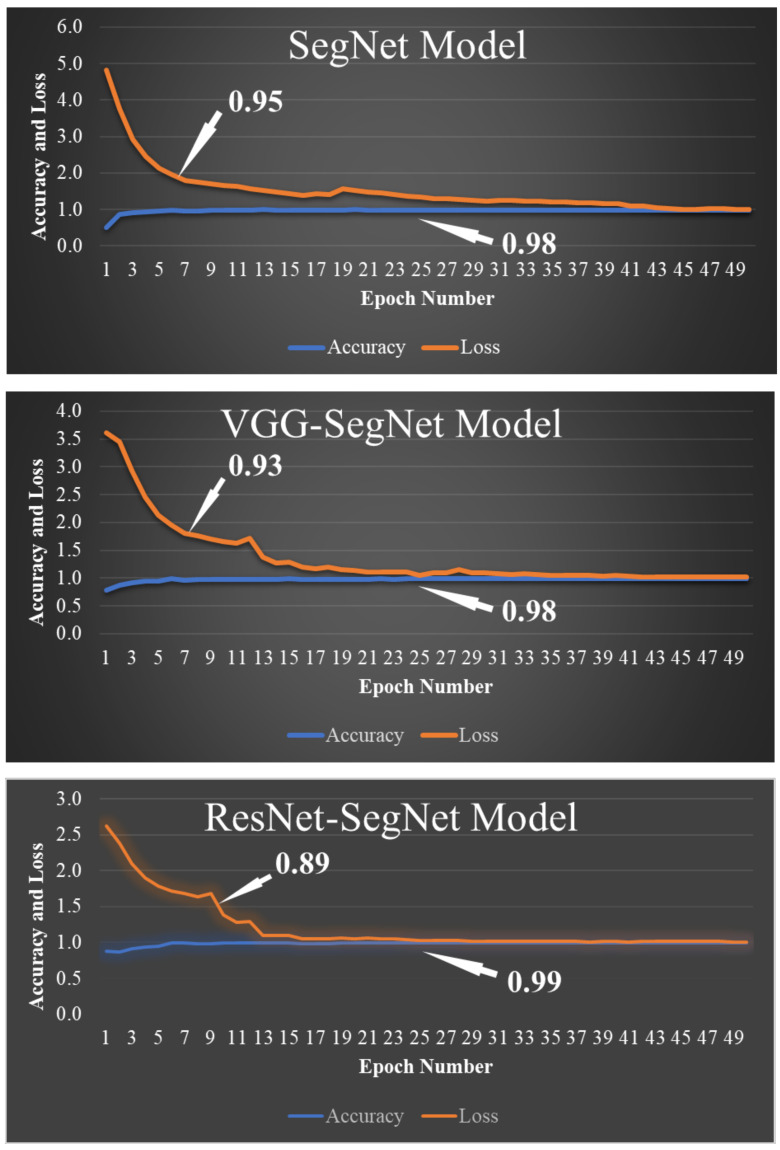
Accuracy and loss from the three AI models SegNet, VGG-SegNet, and ResNet-SegNet.

COVLIAS 1.0 was designed to run 40% training and 60% unseen data sets from 5000 scans. The output of the system reported the binary mask for the left and right lung jointly. These masks were then used for computing the segmented region, given the CT grayscale COVID-19 scans. Figure 9 shows the segmented mask (column 2), segmented lung region (column 3), and overlay image of segmented lung mask of green color over the grayscale image (column 4). Figure 10 shows the accuracy (Equation (4)) and the loss functions (Equation (1)) using the three AI models such as SegNet, VGG-SegNet, and ResNet-SegNet. The accuracy and loss values were then plotted as a stacked line chart to show the increase in the accuracy and decrease in the loss of the training model.

### 4.2. Performance Evaluation

As part of the performance evaluation, three metrics and nine evaluation criteria were considered. First are the visualization comparisons, second is the lung area metrics, and third are the long axis metrics. Section 4.2.1 presents the visualization of boundary error and lung area error (LAE). It has two sets of visualization components (i) visualization of the model boundary against the ground truth boundary with different boundary colors; and (ii) visualization of the superimposition of the model lung region against the GT lung region with different colors; Section 4.2.2 presents the performance evaluation based on the LAE and consists of (i) cumulative plot of the LAE; (ii) Bland–Altman plot for the LAE; (iii) JI and DS for the lung region and (iv) ROC curves for AI-based model performance. Section 4.2.3 presents the performance evaluation based on the lung long axis error (LLAE) and consists of the following components: (i) cumulative plot of the LLAE; (ii) correlation coefficient of the LLAE; and (iii) Bland–Altman plot for the LLAE.

**Figure 11:**
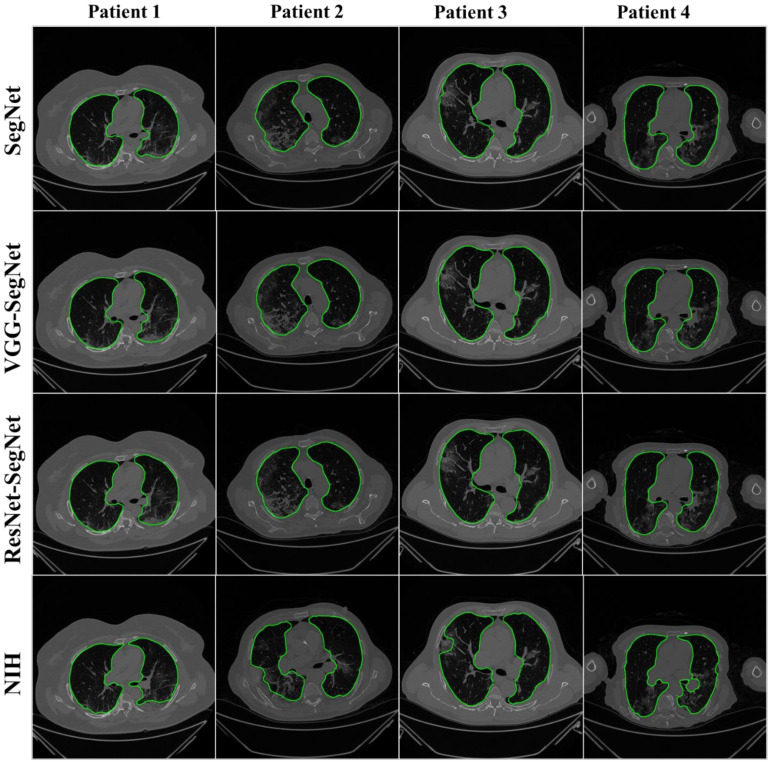
Results from the four AI models and NIH with a grayscale CT slice. Row 1 to Row 4 is four models: SegNet, VGG-SegNet, ResNet-SegNet, and NIH.

The lung masks were then converted into lung boundary images using a region-to-boundary convertor. The lung boundary was superimposed over the original COVID-19 lung CT grayscale scans to visualize the lung, shown in Figure 11. Note that the top three AI models (1 SDL and 2 HDL) performed well (row-1, row-2, and row-3), as can be seen visually, unlike the NIH conventional model (row-4).

#### 4.2.1. Visualization of Lung Boundary and Regional Lung Error

**Figure 12:**
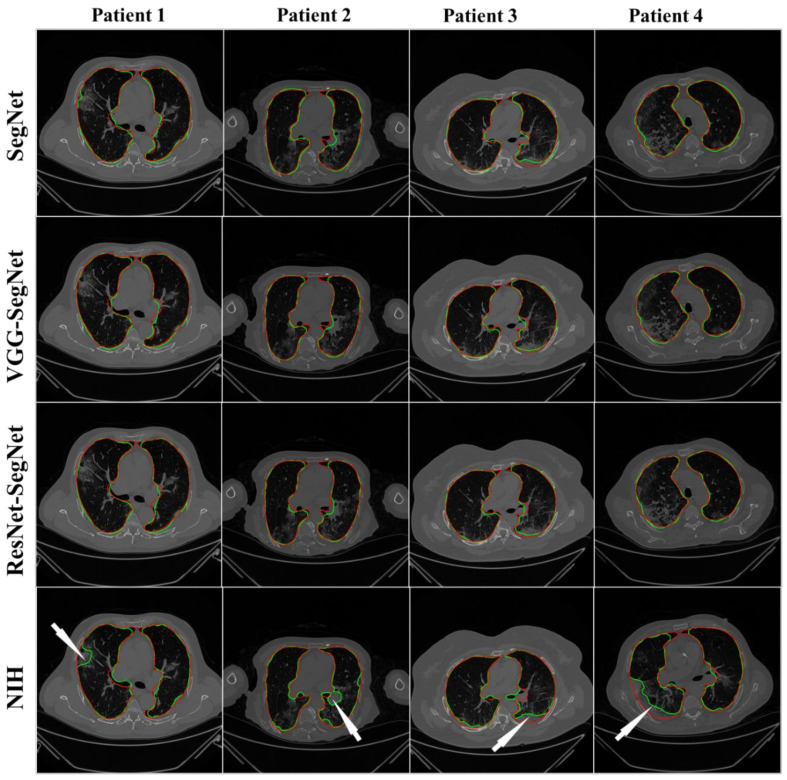
Boundary overlay of the segmented mask from the AI-model (green) vs. GT-mask in the background (red). White arrow shows NIH-segmented boundary not at a right position.

Figure 12 shows the overlay of the AI-model boundary (green) and GT-boundary (red) with a grayscale COVID-19 CT slice in the background. Four models are shown in the rows in the following order: SegNet, VGG-SegNet, ResNet-SegNet, and NIH. As we go down the rows, the boundaries are bumper-to-bumper and show the nearly negligible difference between three AI models and GT, while the pronounced error for the NIH model. The white arrows indicate the region of mismatch between the NIH model and ground truth. The top three rows (SegNet, VGG-SegNet, and ResNet-SegNet) show more closeness between the AI-model borders (green) and the ground truth borders (red).

**Figure 13:**
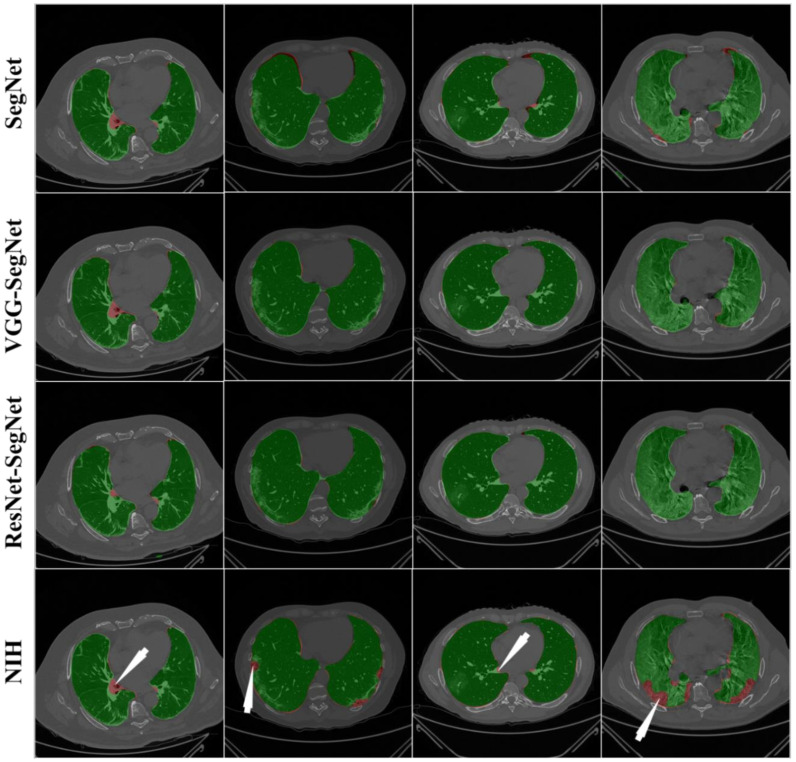
Overlay of the segmented mask from the AI-model (green) vs. GT-mask in the background (red). White arrow shows NIH-segmented boundary not at a right position.

**Figure 14:**
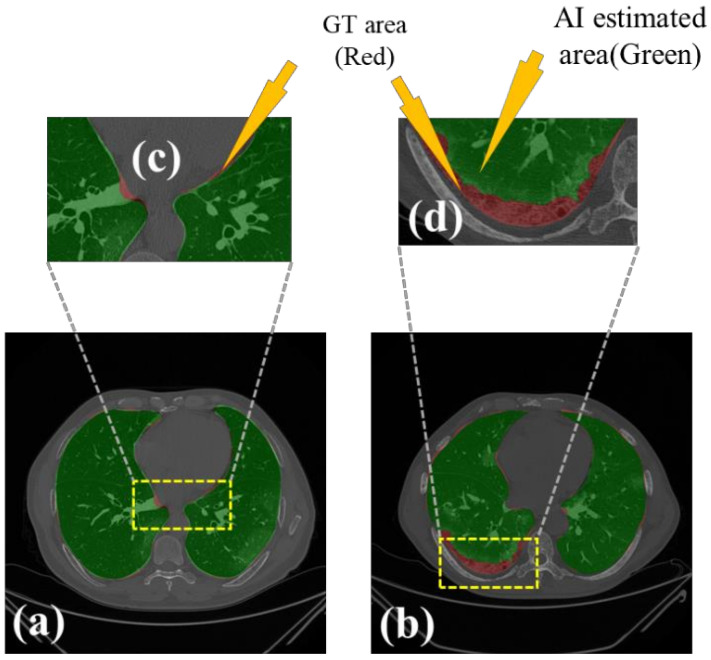
Visualization of the zoomed region-of-interest showing the difference between AI area (green) and GT area (red). (**a**) central lung region, (**b**) basal region of the lung, (**c**) zoom of the central region, and (**d**) zoom of the basal region. Arrow shows the difference between GT and AI area.

**Figure 15:**
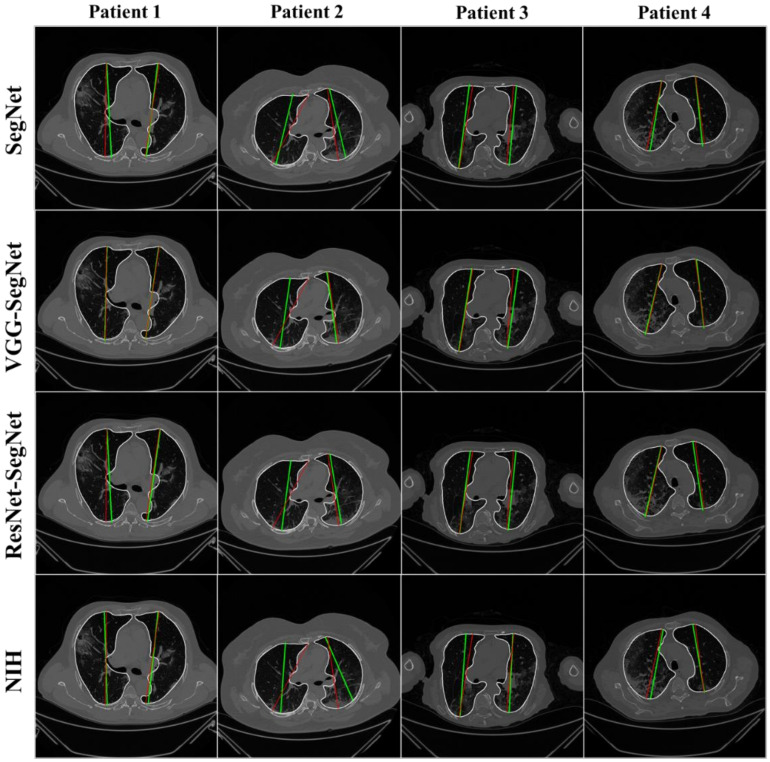
Display of the AI-long axis (green) and GT-long axis (red) showing the reach of the top apical zone and bottom basal zone of the left and right lungs. Rows are the four models, and columns are sample four patients COVID-19 CT scans. White is the GT delineated borders of the lungs by the tracer using ImgTracer(TM).

Figure 13 shows the lung region using AI-mask from the AI-model (green) vs. GT-mask (red). Note that the slight difference is visible between the two-colored masks for the first three AI models, while a more significant difference is seen between the NIH model and GT, typically at the basal region (shown by the white arrows). For better visualization of the differences in area region, we have taken two sample slices in Figure 14, where the combination (c) shows the zoomed version of (a), and combination (d) shows the zoomed version of (b). Figure 15 shows the long axis of the AI-model (green) and GT (red) with a grayscale COVID-19 CT slice in the background. Four models are shown in the rows in the following order: SegNet, VGG-SegNet, ResNet-SegNet, and NIH.

#### 4.2.2. Performance Metrics for the Lung Area Error

##### Cumulative Plot for Lung Area Error

**Figure 16:**
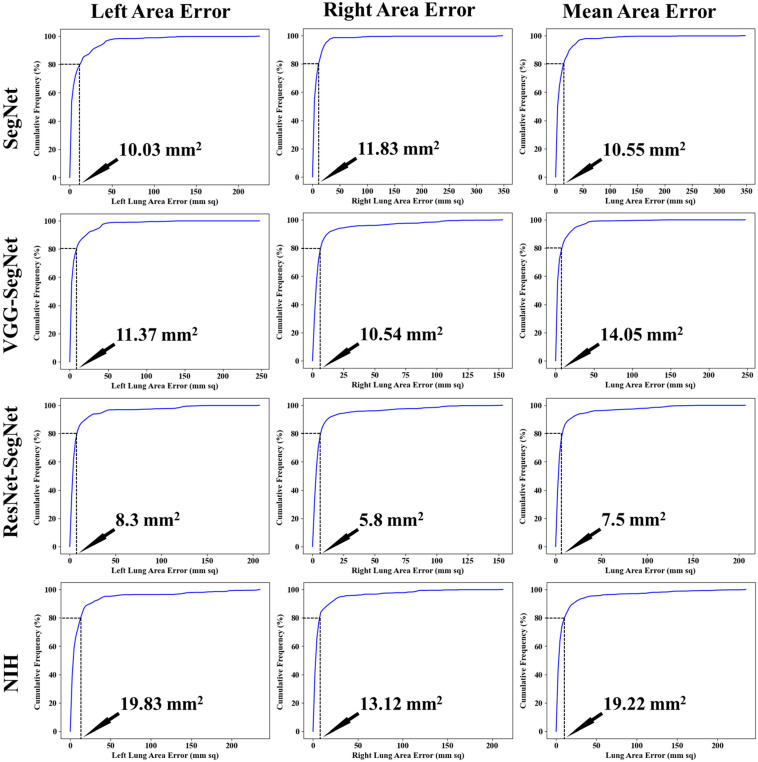
Cumulative plot for LAE for the four models corresponding to left, right, and mean.

Cumulative frequency analysis is the analysis of the frequency of occurrence of the LAE against the reference value. Figure 16 below shows the cumulative distribution of left and right LAE over 3000 images corresponding to the test dataset (60% of 5000 scans). A cumulative frequency threshold of 80% was chosen to compare the four different models: SegNet, VGG-SegNet, ResNet-SegNet, and NIH. Note that, even though the AI models perform exceptionally well, the best of all was the HDL model ResNet-SegNet with 80% LAE for left, right, and combined left/right as 8.3 mm^2^, 5.8 mm^2^, and 7.5 mm^2^, respectively. Furthermore, note that the NIH showed the lowest performance at an 80% threshold (19.83 mm^2^, 13.12 mm^2^, and 19.22 mm^2^, corresponding to the left lung, right lung, and mean of the left lung and right lung). It is also interesting to note that the right LAE was lower than the left LAE for all four models.

##### Lung Area Error

**Figure 17:**
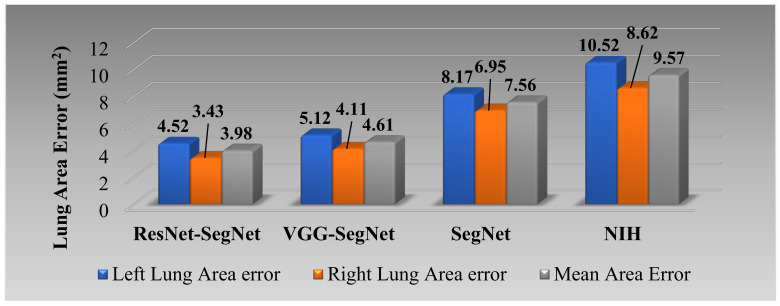
Bar Chart showing the LAE for the four models corresponding to left (blue), right (red), and mean (green) areas. LAE was in the following order ResNet-SegNet < VGG-SegNet < SegNet < NIH.

Figure 17 shows the increasing LAE between the AI-models and GT for the left, right, and mean areas was in the order of ResNet-SegNet < VGG-SegNet < SegNet < NIH. This is consistent with Figure 16, where the LAE increases in the order of ResNet-SegNet < VGG-SegNet < SegNet < NIH, demonstrating that AI models (SDL and HDL models) are superior to NIH conventional models.

##### Correlation Plots for Lung Area

**Figure 18:**
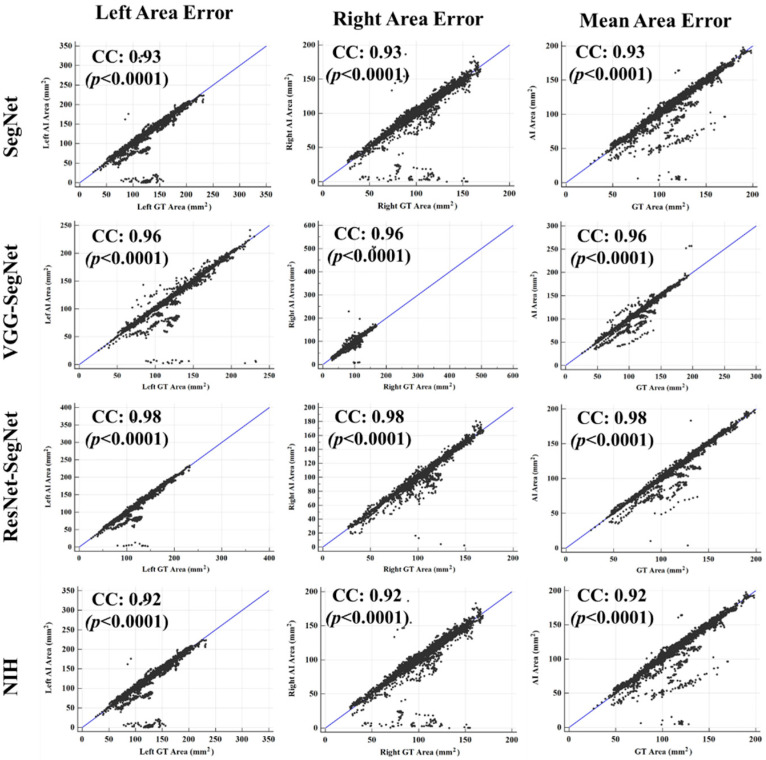
Correlation plot for lung area for four models corresponding to left, right, and mean.

**Figure 19:**
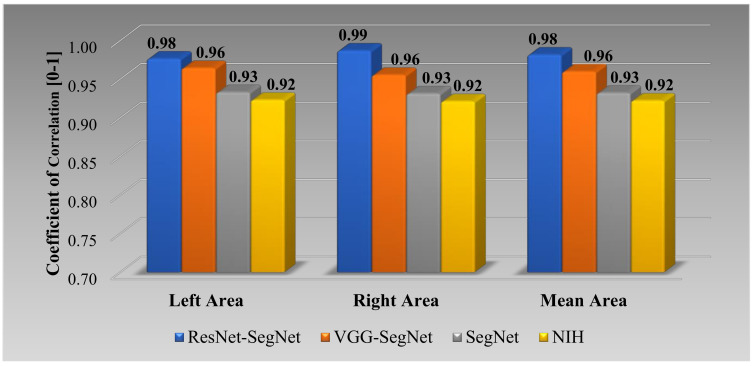
Plot for correlation coefficient between the AI model and GT area for the four types of model: ResNet-SegNet, VGG-SegNet, SegNet, and NIH for left lung, right lung, mean of the two lungs.

Figure 18 (4 × 3 matrix) shows the coefficient of correlation (CC) plot for AI-model lung area vs. GT area using the four models (rows) corresponding to left, right, and mean (columns). The CC values of all the models ranged from **0.91** to **0.98** (*p*-value < 0.0001). The 3D bar plot is shown in Figure 19 for the left, right, and mean lungs. For the left lung, the decreasing order of the CC performance was ResNet-SegNet, VGG-SegNet, SegNet, and NIH. Thus, HDL models were better than the SDL model. This order remained the same for the left lung, right lung, and mean lung area. These results were consistent with Figure 16, Figure 17 and Figure 18.

##### Jaccard Index and Dice Similarity for Lung Area

**Figure 20:**
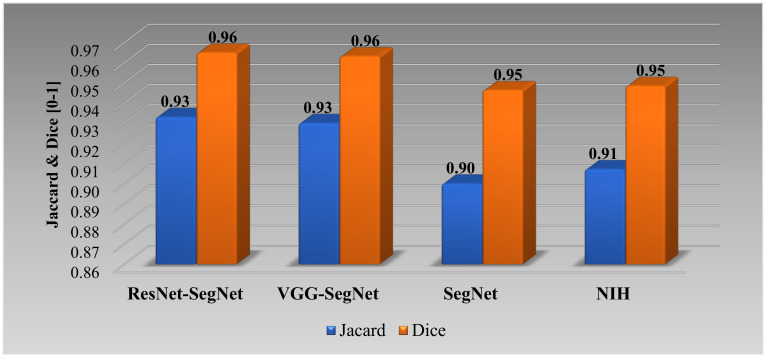
Bar Chart for JI and DS for four (two-hybrid, one deep, and NIH) models.

**Figure 21:**
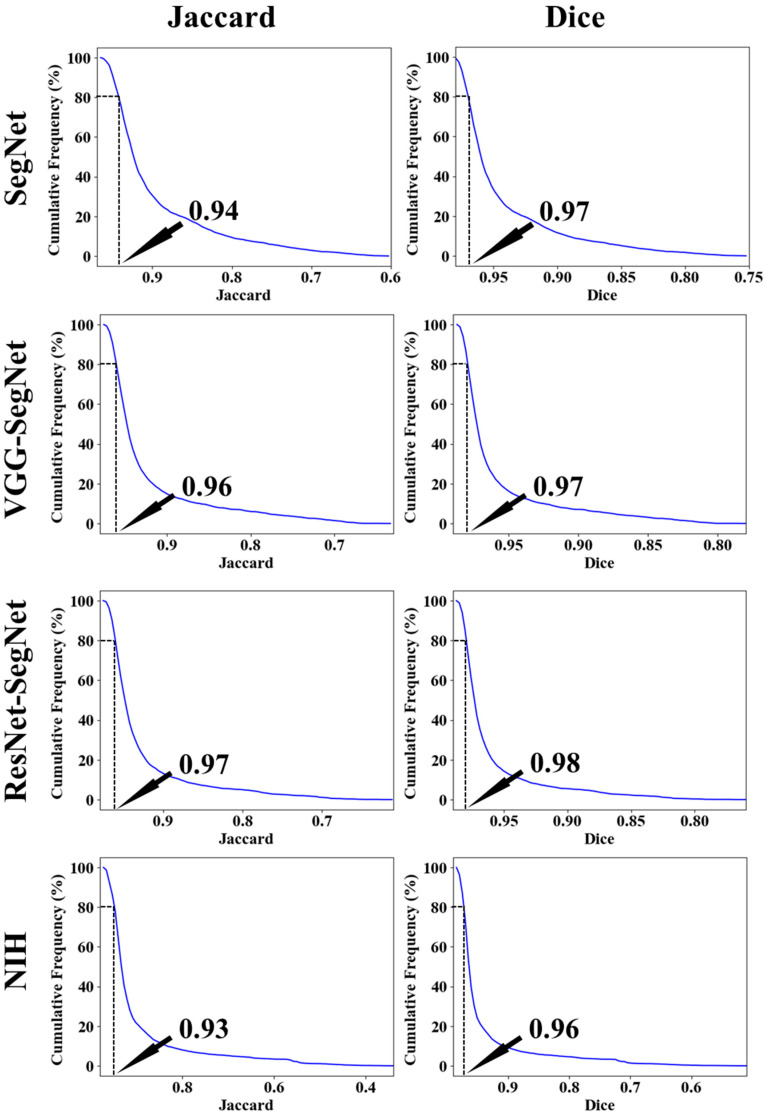
Cumulative frequency for JI and DS for the four models.

Figure 20 below shows the bar chart for the four models using the Jaccard index (JI) (blue) and Dice similarity (DS) (red). The performance of the models (best to worst) had the following order ResNet-SegNet > VGG-SegNet > SegNet > NIH, consistent with Figure 16, Figure 17, Figure 18 and Figure 19. The cumulative frequency for JI and DS for the four models is shown in Figure 21 (left column for JI and right column for DS). Figure 21 shows that 80% of the CT scans had a JI of 0.94, 0.96, 0.97, and 0.93, respectively, for the four models. The DS for 80% of the CT scans had DS of 0.97, 0.97, 0.98, and 0.96, respectively for the four models SegNet, VGG-SegNet, ResNet-SegNet, and NIH conventional models.

##### Bland–Altman Plot for Lung Area

**Figure 22:**
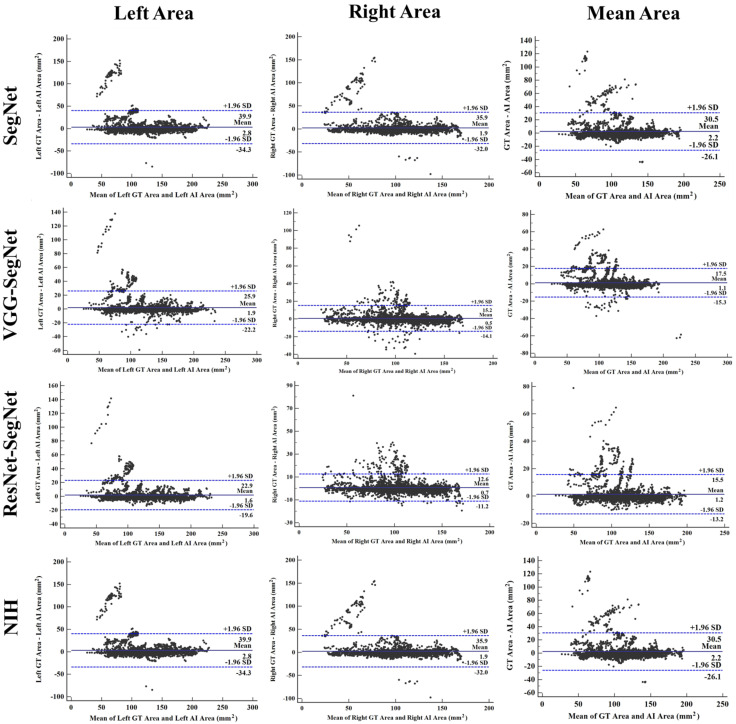
BA plot for LAE for the four models (rows) corresponding to left, right, and mean (columns).

A Bland–Altman plot is used to show the concurrency between two methods using the same variable. We follow the protocol for Bland–Altman computation based on our previous paradigms [40,51]. Figure 22 below shows the Bland–Altman plot by computing (i) mean and (ii) difference between AI model area and GT area corresponding to 3000 COVID-19 test CT scans. The strongest concentration can be seen for the HDL models over the SDL model and NIH models. This is consistent with previous plots.

##### ROC Plots for Lung Area

**Figure 23:**
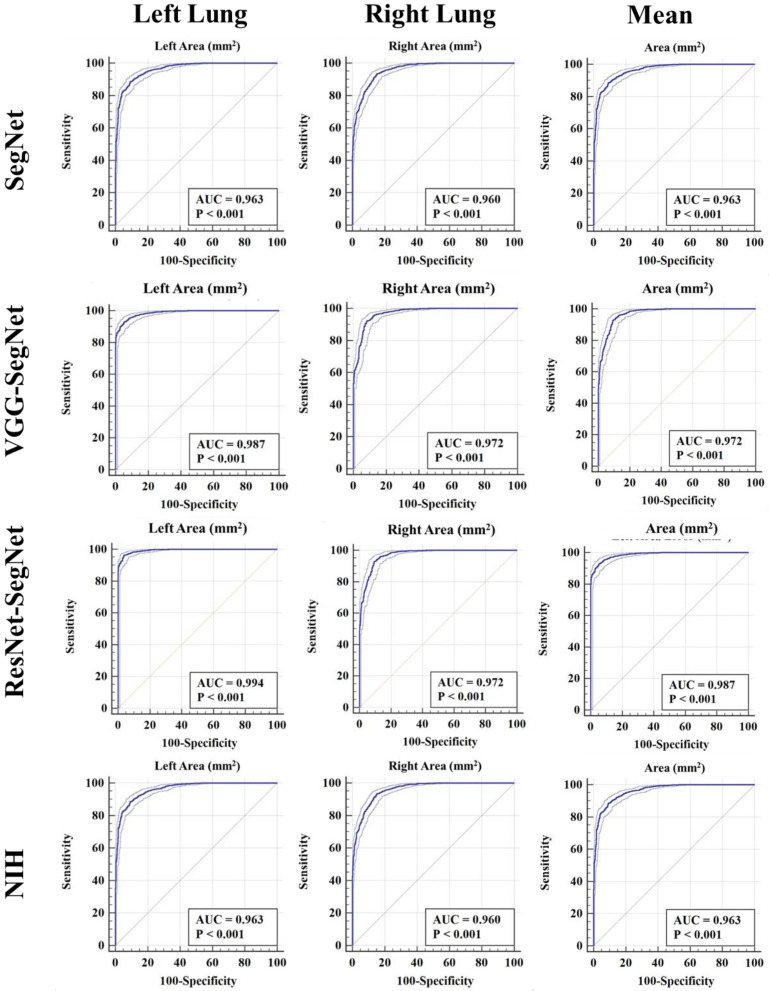
ROC plot for four models corresponding to left, right, and mean.

Figure 23 shows ROC plots for four models (rows) corresponding to left, right, and mean (columns). The corresponding AUC values, along with their *p*-values, are shown as well. All the AUCs were in the range of 0.96 to 0.99 (*p*-value < 0.0001). The highest performance was for ResNet-SegNet for the left, right, and mean lungs and given as **0.994, 0.974**, and **0.987**, respectively. The performance of the models for best to worst had the following order: ResNet-SegNet > VGG-SegNet > SegNet > NIH.

#### 4.2.3. Performance Evaluation Using Lung Long Axis Error

##### Cumulative Frequency Plot for Lung Long Axis Error

**Figure 24:**
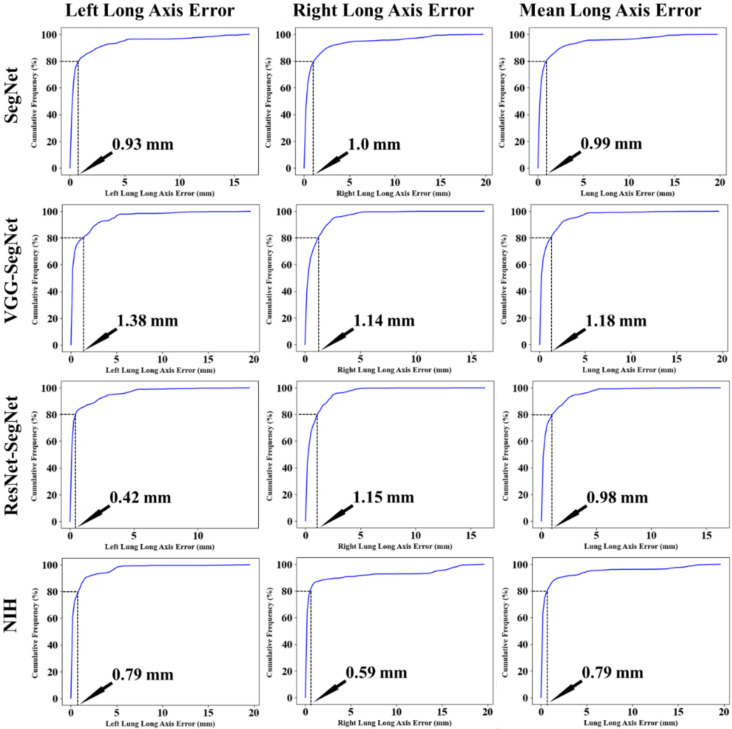
Cumulative plot for LLAE along with an 80% threshold for cumulative frequency for LLAE.

**Figure 25:**
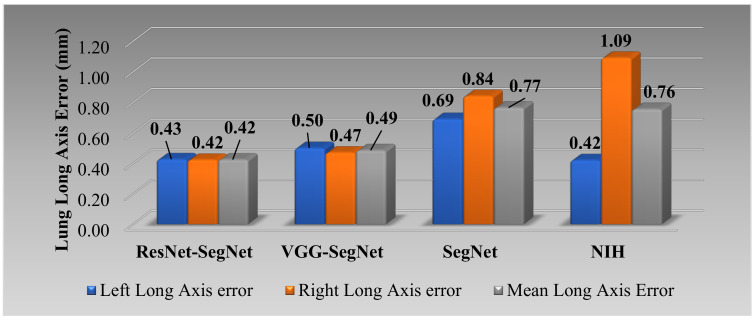
Bar chart for LLAE using four models.

Figure 24 shows the cumulative distribution of left and right LLAE over 3000 images from the test dataset. The cumulative frequency threshold of 80% was chosen to show the comparison amongst the three models. The corresponding bar charts for the LLAE using four models are shown in Figure 25. Decreasing error is shown from right to left, showing the performance of ResNet-SegNet, VGG-SegNet, SegNet, and NIH, which is consistent with area error in Section 4.2.2.

##### Correlation Plot for Lung Long Axis Error

**Figure 26:**
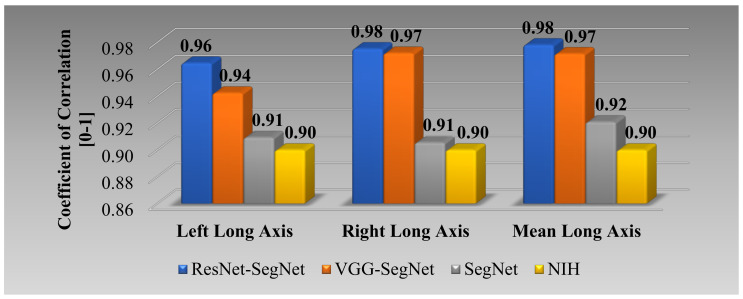
Plot for correlation coefficient between the AI-based LLA vs. GT.

**Figure 27:**
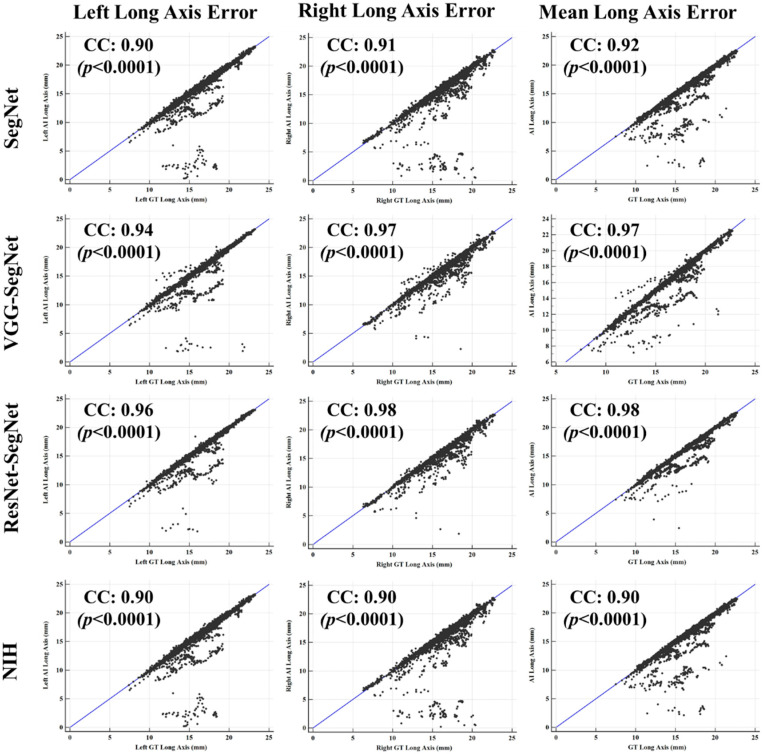
Correlation plot for LLA error for the four models corresponding to left, right, and mean.

Figure 26 shows the correlation plots between lung long axes of the model vs. GT for the left, right, and mean. The CC varied from 0.90 to 0.98 (*p* < 0.0001). The order of the performance was from ResNet-SegNet, VGG-SegNet, SegNet, and NIH. The bar chart plot is shown in Figure 27, and the performance follows the pattern nearly the same order as ResNet-SegNet > VGG-SegNet > SegNet > NIH.

##### Bland–Altman Plots for Lung Long Axis Error

**Figure 28:**
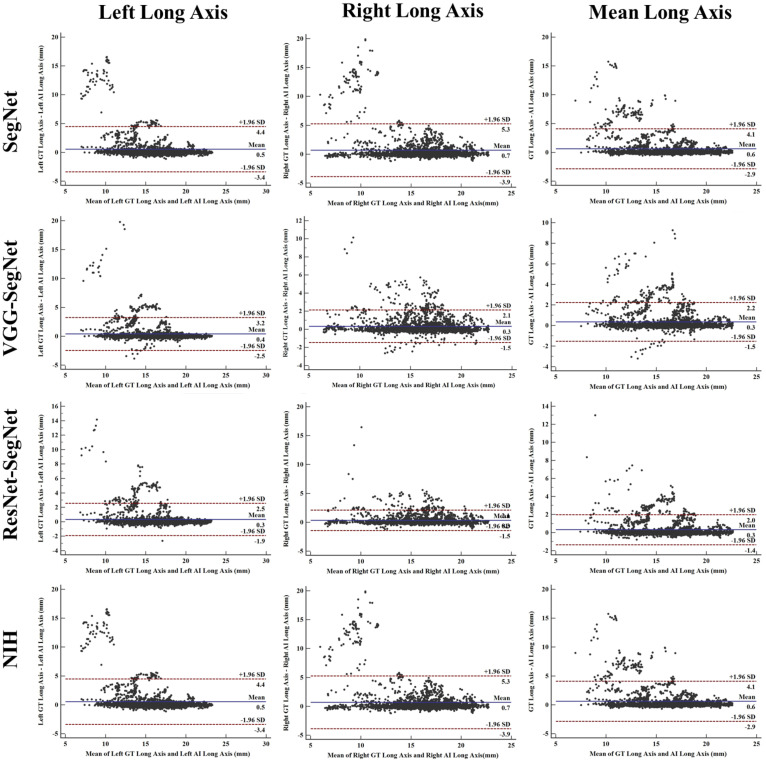
BA plot for LLA for four models corresponding to left, right, and mean.

Figure 28 shows the Bland–Altman (BA) plot for the lung long axis for four models corresponding to left, right, and mean. The order of the performance was ResNet-SegNet, VGG-SegNet, SegNet, and NIH. This can be seen from the spread of the deviations.

##### The Figure of Merit Using Lung Area

The figure of merit (*FoM*) is defined in terms of the central tendency of the error. Let $A_{ai}\left(m,n\right)$ and $A_{gt}\left(n\right)$ represent the lung area using AI model ‘*m*’ and GT, respectively, for the image ‘*n*’. Considering *N* as the total number of CT scans, the corresponding mean AI for model *m* and GT can be represented as ${\bar{A}}_{ai}(m)$ and ${\bar{A}}_{gt}$, as defined in Equation (3), and the *FoM* can be expressed in Equation (5).

Table 1 shows the values for *FoM* for the models against the GT. The order of performance for the models is SegNet, VGG-SegNet, SegNet, and NIH:

(5) $$FoM\left(m\right)=100-\left[\left(\frac{\left|{\bar{A}}_{ai}\left(m\right)-\;{\bar{A}}_{gt}\right|}{{\bar{A}}_{gt}}\right)*100\right],$$

where ${\bar{A}}_{ai}\left(m\right)=\frac{\sum_{n=1}^{N}A_{ai}\left(m,n\right)}{N}$ & ${\bar{A}}_{gt}=\frac{\sum_{n=1}^{N}A_{gt}\left(n\right)}{N}$.

Percentage Improvement of AI Models against the NIH Model Using the Lung Area and Lung Long Axis.

This performance measure shows the improvement of the AI models against the NIH, the so-called benchmarking solution (Table 2).

## 5. Discussion

This study presented a COVLIAS 1.0 system that consisted of two hybrid deep learning and one solo deep learning artificial intelligence model for lung segmentation in COVID-19 CT lung scans. The system was then compared against the conventional NIH-released model. Seventy-two COVID-19 patients were considered for the study, which consisted of 5000 CT scans. Cross-validation protocol was implemented using 40% training and 60% testing data sets. The performance of the system was computed using three main kinds of metrics. This consisted of two sets of visualization components: (i) visualization of the model boundary against the ground truth boundary with different boundary colors; and (ii) visualization of the superimposition of the segmented model lung region against the GT lung region with different colors; the second set of performance evaluation was based on the LAE and consisted of (i) cumulative plot of the LAE; (ii) Bland–Altman plot for the LAE; (iii) JI and DS for the lung region; and (iv) ROC curves for AI-based model performance. The third set of performance evaluation was based on the LLAE and consisted of the following components: (I) cumulative plot of the LLAE; (ii) correlation coefficient of the lung long axis; (iii) correlation coefficient of the lung long axis error; and (iv) Bland–Altman plot for the LLAE. In all the metrics, HDL performed better than SDL and both were better than the conventional NIH model, retaining the order of ResNet-SegNet > VGG-SegNet > SegNet > NIH. Note that the COVILAS 1.0 system (AtheroPoint™, Roseville, CA, USA) was designed using Python language unlike other tools typically designed in MATLAB. The COVLIAS 1.0 used solo deep learning and hybrid deep learning solutions for lung segmentation for CT lung COVID-19 scans. The Python system was developed in a Windows 10 environment, while the graphical user interface was designed using JavaScript. It adapts multitasking and multithreaded architecture for COVLIAS 1.0.

### 5.1. Benchmarking

Several studies have been published with deep learning systems based on chest CT imaging to distinguish COVID-19 cases from non-COVID-19 cases (which may include normal and abnormal cases) and segment the COVID-19 region from the lungs [52,53,54,55]. However, most of them do not include lung segmentation, boundary estimation, or lung region mask overlays compared to GT. Table 3 shows the benchmarking table showing five studies: Priya et al. (2021) [56], Saood et al. (2021) [57], Paluru et al. (2020) [58], and Cai et al. (2020) [59], which focused on lung segmentation. The table also compares against the proposed study by Suri et al. (2021). Cai et al. [59] have used a deep learning-based UNet model to segment the lung and show the boundary of the segmented lung with a DSC of 0.98 and Jaccard of 0.96. Xiao et al. [60] have demonstrated CNN architecture for segmentation of lung and showed a DSC of 0.98. Our study used a hybrid deep learning AI solution for computing lung region with a study database of 5000 CT scans demonstrating DS of 0.97, 0.97, 0.98, and 0.96. The JI for the four models were 0.94, 0.96, 0.97, and 0.93, respectively. The AUC for the four models were 0.96, 0.96, 0.97, and 0.98, respectively, the highest of the previous methods. The accuracies of the proposed AI models were 98%, 98%, and 99%, respectively. Overall, the proposed study offers a first-time hybrid solution in conjunction with solo deep learning and benchmarking against the NIH-based approach, proving our hypothesis that hybrid deep learning artificial intelligence models are superior to solo deep learning models for COVID-19 lung segmentation.

### 5.2. A Special Note on the HDL Model

Our results show that HDL is superior to SDL, and both were superior to the conventional NIH model. HDL takes advantage of combining the two SDL models. Several studies were published in the image classification area that showed HDL was superior to SDL. EL-Kenawy et al. [61] used Stochastic Fractal Search (SFS) combined with the Whale Optimization Algorithm (WOA) as part of the HDL, and the authors showed superior results compared to SDL models such as AlexNet, VGGNet16, VGGNet19, GoogleNet, and ResNet50. Vijayalakshmi et al. [62] implemented an HDL model where the authors combined SVM with VGG16 and showed superior results compared to SDL such as VGG16 or VGG19.

### 5.3. A Note on Comparison between the Proposed AI and NIH Conventional Models

The fundamental difference between the proposed AI models and the NIH model is the role of feature extraction during the lung segmentation process. The feature extraction step is the most vital component of the entire system. This component is accomplished automatically in the AI model, unlike the semi-automatically implemented in the NIH model. This automaticity provides considering time-saving in AI models, unlike in the conventional NIH model. Second, the NIH model has several sub-systems which are user-controlled, such as seed point selection, and image format conversion from High dynamic range (HDR) to Neuroimaging Informatics Technology Initiative (NII/NIfTI) to Portable Network Graphics (PNG) format. It is very tedious and time-consuming when you have to execute this on 5000 CT slices. This is where the AI system comes in handy, is time saving, and is efficient. Our analysis showed that the AI model typically takes one second on any test CT slice, unlike in the NIH model, which took 10 min for a single slice. Note that the NIH model required that the user inputs the entire CT volume of the patient for segmenting the lungs in the CT scans. The processing time for the volume of interest consisting of typically 60 selected slices took ~20 min based on the hardware configuration. This included uploading of volumes, semi-automated segmentation, format conversion, and saving of all the segmented results.

### 5.4. Strength, Weakness, and Extension

The study presented two HDL models, one SDL model, and one conventional NIH model for lung segmentation in COVID-19 CT lungs for a cohort of 5000 images. The results of the study showed consistency where HDL models were superior to SDL models. The online system took less than 1 s.

Even though the COVLIAS 1.0 was the first of its kind where HDL was designed and developed, more validation is needed for conduction involving multicenter studies [39,63]. Furthermore, cyclic cross-validation needs to be conducted for further comparisons [64,65]. The system can be extended to estimate COVID-19 severity in the lung region while keeping the services cost-effective [66]. Variability analysis can also be conducted where multiple tracers can be involved [40]. The COVLIAS 1.0 is a pilot study with encouraging results. We intend to pursue more CV protocols with large data size and more models in the big data framework [67] in the future.

Comorbidity is an essential factor that has been linked to COVID-19 right from the beginning. Recently, a study was conducted that showed the relation of the effect of the comorbidity on AI model design [6]. In our study, the patients had no prior issues before getting admitted to Novara hospital, Italy during a pandemic. In some cases, however, it is possible that the patients had concomitant or incidental findings such as scars or small nodules. The scope of this study did not incorporate such incidental findings enough to incorporate them into the AI model. This could potentially be a future possibility of more sophisticated data collection and modeling of AI. Furthermore, the COVLIAS 1.0 system can be extended to other kinds of pneumonia, interstitial lung diseases, and cancer [25,68]. Lastly, the AI model can be compared with conventional models as previously attempted applications [69].

## 6. Conclusions

The study presented four kinds of lung segmentation methods for COVID-19 CT lung scans under the COVLIAS 1.0 (AtheroPoint™, Roseville, CA, USA). It consisted of three AI-based models: two-hybrid deep learning (HDL) models and one SDL. These three AI models were benchmarked against the NIH-based conventional model. The COVILAS 1.0 used a database of 5000 COVID-19 CT images for designing the cross-validation protocol that consisted of 4:6 ratios for training to testing with validation data.

Comprehensive performance matrices were designed such as (i) visualization of the model boundary against the ground truth boundary with different boundary colors; (ii) visualization of the superimposition of the model lung region against the GT lung region with different colors; (iii) performance evaluation based on the LAE and consisted of (a) cumulative plot of the lung area error; (b) Bland–Altman plot for the lung area error; (c) Jaccard index and dice similarity for the lung region; and (d) ROC curves for AI-based model performance. (iv) performance evaluation based on the lung long axis error and consists of the following components (a) cumulative plot of the lung long axis error; (b) correlation coefficient of the lung long axis error; and (c) Bland–Altman plot for the lung long axis error.

We further conclude that the AI-based models were better than the conventional NIH model, and HDL was superior to SDL. The order of performance for all metrics were ResNet-SegNet > VGG-SegNet > SegNet > NIH. The COVLIAS 1.0 system is currently under evaluation by radiology departments at friendly sites.

## Figures and Tables

**Table 1:** *FoM* for the segmentation modes for lung area and lung long axis.

	Lung Area			Lung Long Axis		
	Left	Right	Mean	Left	Right	Mean
SegNet	94.28	97.6	95.94	97.03	98.53	97.78
VGG-SegNet	99.09	99.56	99.32	99.7	99.61	99.65
ResNet-SegNet	99.03	99.98	99.55	99.72	99.62	99.67
NIH	94.98	98.69	96.83	97.76	96.09	96.92

**Table 2:** Improvement of three AI models against the NIH model for lung area and lung long axis.

	Lung Area Error			Lung Long Axis Error		
	Left Lung	Right Lung	Mean	Left Lung	Right Lung	Mean
ResNet-SegNet	57%	60%	58%	42%	44%	43%
VGG-SegNet	51%	52%	52%	31%	37%	34%
SegNet	22%	19%	21%	5%	13%	4%

**Table 3:** Benchmarking table.

Author (Year)	# of Patients	Demo.	# of Images	IS^2^	# of Tracers	AI Model	SDL vs. HDL	Dim.	Training Speed	Heatmap	VS	AE	BE	DS	JI	AUC	ACC (%)
Priya et al. (2021) [56]	-	No	78	-	-	Squeeze Net	HDL	2D	-	Yes	No	-	Yes	-	-	-	-
Saood et al. (2021) [57]	-	No	100	256^2^	-	UNet, SegNet	SDL	2D	-	-	No	-	-	0.73, 0.74	-	0.94, 0.95	91, 95
Paluru et al. (2020) [58]	69	No	4339	512^2^	-	Anam-net	SDL	2D	27 min	-	Yes	-	-	0.75	-	0.99	99.1
Cai et al. (2020) [59]	99	Yes	6301		2	UNet	SDL	2D	-	yes	No	-	-	0.98	0.96	-	-
Suri et al. (2021)	72	Yes	5000	768^2^	1	NIH, SegNet, VGG-SegNet, ResNet-SegNet	SDL and HDL	2D	18 min	No	No	Yes	Yes	0.96, 0.97, 0.97, 0.98	0.93, 0.94, 0.96, 0.97	0.96, 0.96, 0.97, 0.98	NA, 98, 98, 99

Demo.: Demographics, VS: Variability studies; IS^2^: Image size (2 represents dimension along *x*-axis & *y*-axis); AE: Area error; Dim.: Dimensionality of the image (2D vs. 3D); BE: Boundary error; DS: Dice similarity; JI: Jaccard index; AUC: Area under the curve; SDL: Solo deep learning; HDL: Hybrid deep learning; ACC: Accuracy.

## Data Availability

Not applicable.

## Acronym Table

**SN**	**Symbol**	**Description of the Symbols**
1	ACC	Accuracy
2	AE	Area Error
3	AI	Artificial Intelligence
4	ARDS	Acute Respiratory Distress Syndrome
5	AUC	Area Under the Curve
6	BA	Bland–Altman
7	BE	Boundary Error
8	CC	Correlation coefficient
9	CE	Cross Entropy
10	COVID	Coronavirus disease
11	COVLIAS	COVID Lung Image Analysis System
12	CT	Computed Tomography
13	DL	Deep Learning
14	DS	Dice Similarity
15	FoM	Figure of merit
16	GT	Ground Truth
17	HDL	Hybrid Deep Learning
18	IS	Image Size
19	JI	Jaccard Index
20	LAE	Lung Area Error
21	LLAE	Lung Long Axis Error
22	NIH	National Institute of Health
23	PC	Pixel Counting
24	RF	Resolution Factor
25	ROC	Receiver operating characteristic
26	SDL	Solo Deep Learning
27	VGG	Visual Geometric Group
28	VS	Variability studies
29	WHO	World Health Organization

## Symbol Table

**SN**	**Symbol**	**Description of the Symbols**
1	$L_{CE}$	Cross Entropy-loss
2	$L_{DSC}$	Dice Similarity Coefficient-loss
3	*m*	Model number used for segmentation in the total number of models M
4	*n*	Image scan number a total number of image N.
5	${\bar{A}}_{ai}\left(m\right)$	Mean estimated lung area for all images using AI model ‘*m*’
6	$A_{ai}\left(m,n\right)$	Estimated Lung Area using AI model ‘*m*’ and image ‘*n*’
7	$A_{gt}\left(n\right)$	GT lung area for image ‘*n*’
8	${\bar{A}}_{gt}$	Mean ground truth area for all images N in the database
9	$FoM\left(m\right)$	Figure-of-Merit for segmentation model ‘*m*’
10	JI	Mean Jaccard Index for a specific segmentation model
11	DSC	Dice Similarity Coefficient for a specific segmentation model
12	$N_{p}$	Sample size required computed using power analysis
13	MoE	Margin-of-Error
14	TP, TN	True Positive and True Negative
15	FP, FN	False Positive and False Negative
16	y_i_	GT label
17	a_i_	SoftMax classifier probability
18	Y_p_	Ground truth image
19	$\hat{Y_{p}}$	Estimated image
20	P	Total number of pixels in an image in *x,y*-direction
21	z	Z-score from standard *z*-table
22	K5-r46	Cross-validation protocol with 40% training and 60% testing
**Deep Learning Segmentation Architectures**		
23	SegNet	SDL model for lung segmentation with reduced learning parameters
24	VGG-SegNet	HDL model designed by fusion of VGG-19 and SegNet architecture
25	ResNet-SegNet	HDL model designed by fusion of ResNet-50 and SegNet architecture
**Conventional Model**		
26	NIH	National Institute of Health segmentation model
